# A case highly suspicious of isolated cardiac sarcoidosis

**DOI:** 10.1007/s12471-016-0837-3

**Published:** 2016-05-11

**Authors:** M. P. Huitema, M. J. Swaans, J. C. Grutters, M. C. Post

**Affiliations:** 1 Department of Cardiology, St. Antonius Hospital, Nieuwegein, The Netherlands; 2 Department of Pulmonology, St. Antonius Hospital, Nieuwegein, The Netherlands

**Fig. 1:**
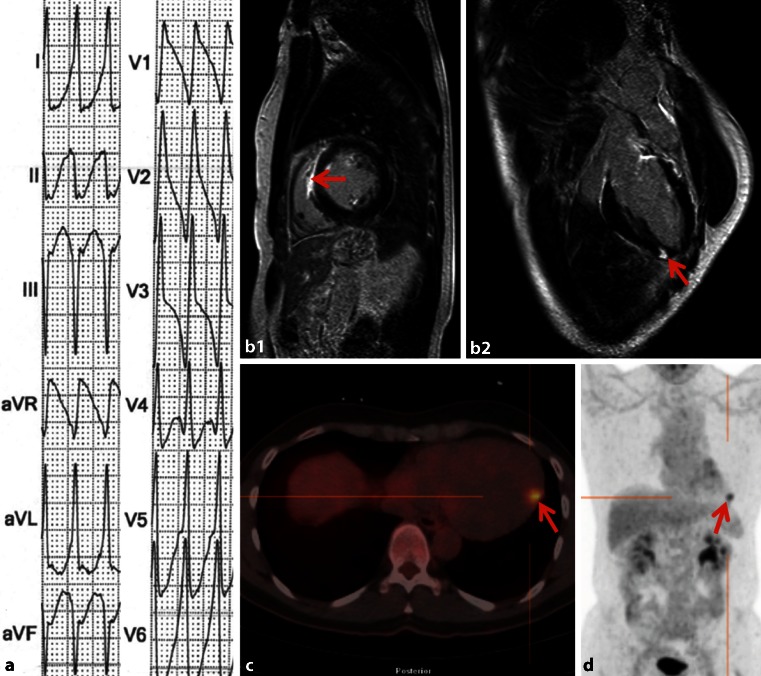
**a** Electrocardiogram showing a broad-complex tachycardia of 240 beats/min, retrograde P waves and right-axis deviation, suggesting a right ventricular origin. Cardiac MRI shows extensive late enhancement at the right ventricular part of the interventricular septum (*arrow* in **b1 and b2**), and a focal lesion in the epicardial inferolateral wall (*arrow* in **c**). **d** FDG PET-CT shows a high uptake lesion of the inferolateral wall (*arrow*), correlating with MRI

A 55-year-old Caucasian male without cardiac history presented with a broad-complex tachycardia of a right ventricular origin (Fig. 1a). After successful electrocardioversion, echocardiography and coronary angiogram showed no significant abnormalities. Electrophysiological examination suggested a right ventricular mid-septal origin of the arrythmia, closely to the HIS bundle. Cardiac MRI (Fig. 1b,c) showed extensive late enhancement at the right ventricular part of the interventricular septum (Fig. 1b) and a focal lesion in the epicardial inferolateral wall (Fig. 1c), both showing high uptake on FDG PET-CT (Fig. 1d). In the laboratory findings, soluble interleukin-2 receptor (sIL-2R), a marker for sarcoidosis, was elevated (5276 pg/ml, normal value <3000 pg/ml), making cardiac sarcoidosis highly likely. FDG PET-CT and examination of skin and eyes showed no signs of extracardiac sarcoidosis and pulmonary sarcoidosis was excluded by high-resolution CT and bronchoalveolar lavage, suggesting a case of isolated cardiac sarcoidosis. The prevalence of cardiac involvement in sarcoidosis varies from 5 % in symptomatic patients to up to 30 % in autopsies [1]. Isolated cardiac sarcoidosis is rare and only described in case series [2]. For diagnosis, multimodality imaging is recommended, including MRI and FDG PET [3]. FDG PET is the modality of choice to examine (extra)cardiac sarcoidosis [4]. Giant cell myocarditis can be considered in the differential diagnosis. However the scan results, elevated sIL-2R, young age and dysrhythmia are typical for cardiac sarcoidosis. A two-chamber implantable cardioverter defibrillator was implanted and prednisolone treatment was started. FDG PET-CT after 3 months showed complete normalisation. Although extremely rare, this case illustrates the possible occurrence of mono-organ localisation of sarcoidosis.
